# Hedgehog signaling controls mouth opening in the amphioxus

**DOI:** 10.1186/s40851-021-00186-8

**Published:** 2021-12-24

**Authors:** Guangwei Hu, Guang Li, Yiquan Wang

**Affiliations:** 1 Jiangsu Key Laboratory of Marine Bioresources and Environment /Jiangsu Key Laboratory of Marine Biotechnology, School of Marine Science and Fisheries, Jiangsu Ocean University, Lianyungang, 222005 China; 2 Co-Innovation Center of Jiangsu Marine Bio-industry Technology, Jiangsu Ocean University, Lianyungang, 222005 China; 3 State Key laboratory of Cellular Stress Biology, School of Life Sciences, Xiamen University, Xiamen, 361102 China

**Keywords:** Hedgehog signal, Mouth opening, *Smo*, Amphioxus

## Abstract

**Introduction:**

The left-sided position of the mouth in amphioxus larvae has fascinated researchers for a long time. Despite the fundamental importance of mouth development in the amphioxus, the molecular regulation of its development is almost unknown. In our previous study, we showed that *Hh* mutation in the amphioxus leads to no mouth opening, indicating a requirement of Hh signaling for amphioxus mouth formation. Nevertheless, since the *Hh* mutant also exhibits defects in early left-right (LR) patterning, it remains currently unknown whether the loss of mouth opening is affected directly by Hh deficiency or a secondary effect of its influence on LR establishment.

**Results:**

We demonstrated that knockout of the *Smo* gene, another key component of the Hh signaling pathway, in the amphioxus resulted in the absence of mouth opening, but caused no effects on LR asymmetry development. Upregulation of Hh signaling led to a dramatic increase in mouth size. The inability of *Smo* mutation to affect LR development is due to *Smo*’s high maternal expression in amphioxus eggs and cleavage-stage embryos. In *Smo* mutants, *Pou4* and *Pax2/5/8* expression at the primordial oral site is not altered before mouth opening.

**Conclusions:**

Based on these results and our previous study, we conclude that Hh signal is necessary for amphioxus mouth formation and that the Hh-mediated regulation of mouth development is specific to the mouth. Our data suggest that Hh signaling regulates mouth formation in the amphioxus in a similar way as that in vertebrates, indicating the conserved role of Hh signaling in mouth formation.

**Supplementary Information:**

The online version contains supplementary material available at 10.1186/s40851-021-00186-8.

## Background

Mouth development in animals has fascinated researchers for decades. In most protostomes, the mouth is derived directly from the blastopore. In deuterostomes (including echinoderms, hemichordates and chordates), however, the mouth is thought to develop independently from the blastopore. The first opening formed by the blastopore becomes the organism’s anus, while the mouth is formed secondarily on the opposite side by perforation of the outer epithelium and the wall of the gut.

Among all living deuterostomes, the amphioxus is an exception with respect to mouth formation, in which the mouth initially opens on the left side. Before amphioxus mouth formation, a population of compact mesoderm cells (also called oral mesovesicle, OMV) is present at the posterior end of the first left somite. As development continues, the dorsal group of these cells develops into Hatschek’s nephridium, while the ventral group becomes interposed between the ectoderm and endoderm in the region where the mouth will soon form [1, 2]. Mouth penetration occurs between the epidermis and remnant of the OMV [1, 2]. The peculiar left-sided mouth in the amphioxus is a long-standing conundrum, and much effort has been devoted to homologizing the amphioxus mouth to that of vertebrates and other deuterostomes [1–9]. Despite the fundamental importance of mouth development in the amphioxus, the molecular mechanisms regulating mouth development in the amphioxus are far less clear. At present, Nodal-Pitx and Bmp signaling pathways have been reported to be associated with mouth formation [2, 10, 11], and inhibition of Nodal or Bmp signaling results in the loss of left-sided identity, leading to the absence of the mouth, suggesting that these two left-right regulatory pathways do not directly control mouth opening in the amphioxus. A paper by Annona et al. (2017) showed that nitric oxide is an essential cell-signaling molecule for amphioxus mouth formation, which provides the first data for directly revealing a molecular mechanism in amphioxus mouth formation [12]. Nevertheless, it is still necessary to consider other signaling pathways during amphioxus mouth morphogenesis.

The Hedgehog (Hh) signaling pathway is one of the key pathways that is essential for metazoan embryonic development. Its involvement in mouth development has been reported in frog: blocking Hh signaling with the chemical inhibitor cyclopamine or SANT1 resulted in the loss of primary mouth opening [13]. Our previous result showed that Hh loss-of-function resulted in failure of mouth formation in the amphioxus, indicating that the Hh signal may be involved in the regulation of amphioxus mouth development [14]. However, since Hh deficiency also led to defects in left-right patterning, it remains unclear whether the absence of mouth formation in *Hh* mutants is a direct consequence of Hh perturbation or a secondary effect of impaired left-right patterning.

In this report, we first compared the expression patterns of *Smo* and *Hh* genes in the amphioxus *Branchiostoma floridae* and then investigated whether Hh signaling in the amphioxus directly controls mouth formation by TALEN-based knockout. Furthermore, we investigated the role of Hh signaling during amphioxus mouth formation by examining mouth marker genes expression. Together, these findings indicate that Hh signaling plays a critical role during mouth formation in the amphioxus and points to conservation of this pathway in regulating mouth development.

## Material and methods

### Experimental animal

Amphioxus *Branchiostoma floridae* were originally acquired from Dr. Jr-Kai Yu (Institute of Cellular and Organismic Biology, Academia Sinica, Taipei, Taiwan), and colonies were maintained in a laboratory culture system as described in the previous report [15]. Thermal induction spawning was performed according to previous report (from 22 °C to 26 °C) [16]. Egg fertilization and embryo culture at 26 °C were carried out according to previous description [17]. Embryos and larvae at the required developmental stages were fixed with 4% PFA in MOPS buffer (pH 7.4) overnight at 4 °C. All embryos were staged according to previously described methods [18].

### Mutant generation and genotyping

*Smo* gene knockout amphioxus was generated using the TALEN method. In brief, TALEN pairs recognizing the coding sequence of the *Smo* gene (Fig. 2a) were designed and assembled according to our previous description [19]. The final TALEN expression plasmids were linearized by *Sac*I restriction enzyme digestion. TALEN mRNA was synthesized using the mMESSAGE mMACHINE T3 kit (Ambion).

TALEN mRNA was microinjected into the egg of the amphioxus followed by fertilization. One day after injection, genomic DNA from injected embryos was isolated and used as the template for PCR. PCR products were digested by the restriction enzyme *BamH*I to estimate the somatic mutation ratio. To obtain germline mutations, TALEN-injected embryos (F0 progeny) were raised to adulthood and outcrossed with wild-type amphioxus. Mosaic founder animals were spawned to generate F1 heterozygotes using PCR and sequencing to detect, characterize and follow mutant alleles as described previously [20]. Homozygous mutants were generated by crossing heterozygous animals.

### Whole-mount in situ hybridization (WISH)

The RNA probes used in this study were amplified using the primers listed in Table 1. The cDNA template for PCR was derived from total RNA extracted from mixed embryos and larvae. PCR products were recombined with the pGEM-T Easy vector (Promage, USA) and transformed into *E. coli*. After sequencing verification, we synthesized digoxigenin DIG-labeled antisense probes for the above genes using SP6 or T7 RNA polymerase (depending on insert orientation). Whole-mount in situ hybridization was performed according to the previously described protocol [21] with slight modifications as follows: the duration of proteinase K treatment varied from 3 to 10 min depending on embryonic stage, and probe incubation was performed at 65 °C overnight.

**Table 1:** List of primers used in this study

Name of gene	Forward primer (5′ → 3′)	Reverse primer (5′ → 3′)	Restriction site
*Smo*	GGTACCTTTCCACCATGTTGAGGAGCG	ACTAGTGGTTCTTCACAGTACTCTGTATC	KpnI/SpeI
*Cer*	GGTACCATGAAGACGAGCGTGAGGAGC	ACTAGTTCAGAAGTACTTATCCCCACATG	KpnI/SpeI
*Nodal*	GGTACCGCAGGCCGAGACCAACACCGC	ACTAGTCTACTGACAGCCGCATTCATCC	KpnI/SpeI
*Lefty*	CTCGAGTACGATGAAACCTGTTCTAGTT	ACTAGTTTACTGTGTGCACGCACACTG	XhoI/SpeI
*Pitx*	GGTACCACATATCTAAGGAGGACATCGTG	ACTAGTTCTTTAGCAAACAAATCCCATACGC	KpnI/SpeI
*Ptch*	ACGGTTGGACATATTCTGTTGC	TGATACCATCCGCTCATTTCTG	NA
*Pou4*	GGTACCAGAACAGATGATGAACGGGAAAC	ACTAGTTTGGGCGGTGCGATAGTAGAG	KpnI/SpeI
*Pax2/5/8*	ATGGACAGGATGACCACGATG	GTGAGAAGAGAAGAAGTTGCC	NA
*Frzb1*	GGTACCGCGATATTGAATTTAGCGTGGT	ACTAGTCGAGTTGTCAGGGTCTTAGCA	KpnI/SpeI

### In vitro mRNA synthesis

The coding sequence of the *Hh* gene was amplified from a cDNA library based on amphioxus embryos. PCR product was cloned into the pXT7 vector. Plasmid DNA was prepared using Plasmid Mini Kit (Omega), linearized with restriction enzyme, extracted by phenol-chloroform and dissolved in RNase-free water. In vitro mRNA synthesis was conducted using T7 mMESSAGE mMACHINE kit (Ambion).

## Results and discussion

**Fig. 1:**
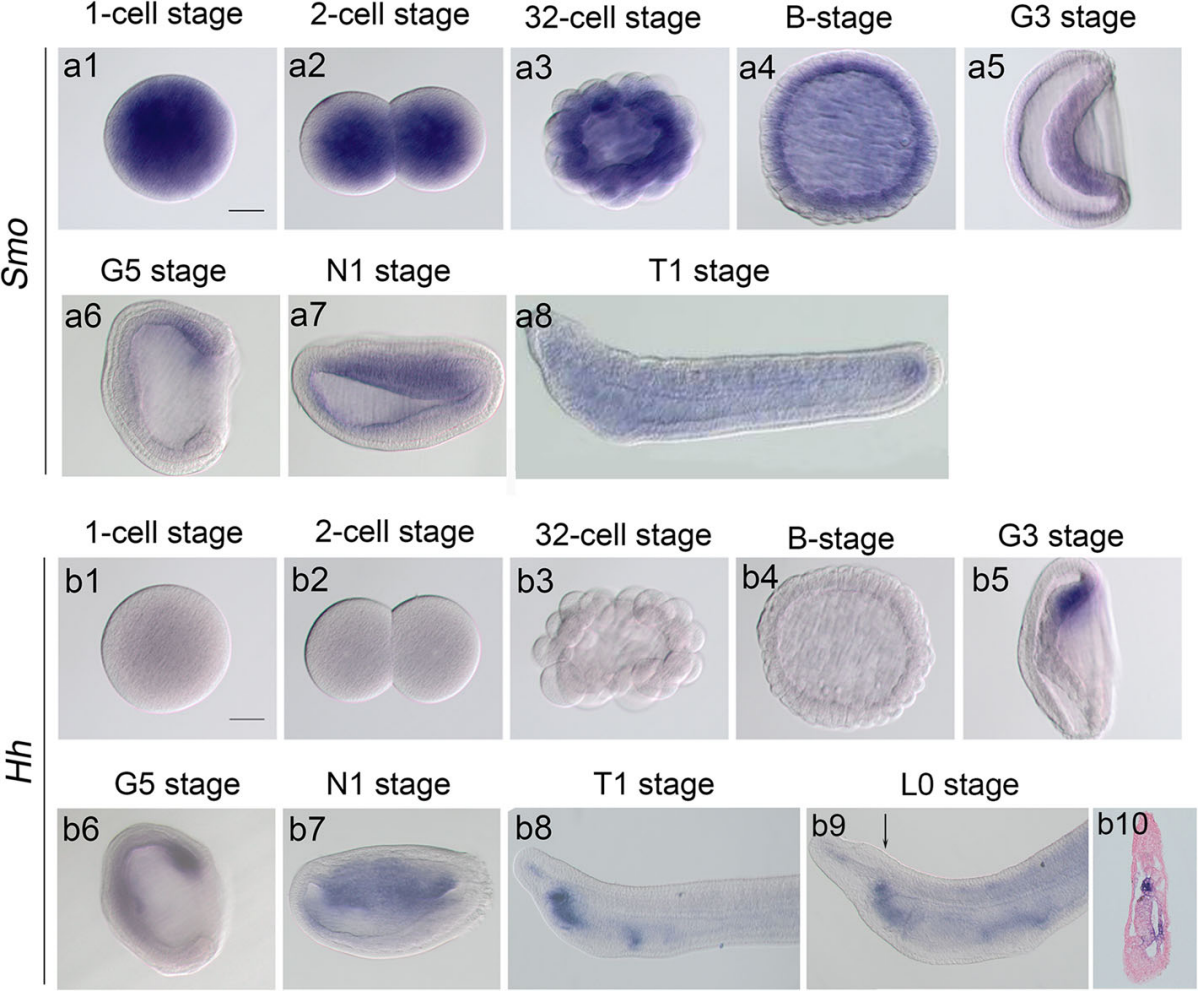
The expression of *Smo* and *Hh* genes in developing amphioxus embryos. a1-a8: Expression patterns of *Smo* at eight stages of amphioxus development. b1-b10: Expression patterns of *Hh* at nine stages of amphioxus development. *Smo* exhibits strong maternal expression in fertilized eggs and early cleavage embryos. *Hh* gene shows zygotic but no maternal expression. Arrow in b9 indicate the section plane in b10. Scale bar, 50 μm

In our previous study, we showed that loss of Hh activity by *Hh* knockout resulted in abnormal left-right patterning and absence of mouth formation in the amphioxus [14]. This raised the possibility that the absence of mouth formation might be a secondary effect of impaired left-right patterning but not directly controlled by Hh signaling. To address this question, we examined the function of *Smo* in amphioxus mouth formation. *Smo* is a receptor and a positive regulator of the Hh signaling pathway in flies and vertebrates [22], and *Smo* gene knockout would theoretically lead to inactivation of the Hh signaling pathway in the amphioxus. Before performing the *Smo* mutation experiment, we first analyzed *Smo* and *Hh* genes expression during several stages of amphioxus embryos with the whole-mount in situ hybridization (WISH) method. We found that *Smo* exhibited strong maternal expression in fertilized eggs and early cleavage embryos (Fig. 1a1-a4). Zygotic expression of *Smo* was first detected at the G5 stage in the dorsal-lateral endomesoderm (Fig. 1a5). In N1 and T1 embryos, *Smo* was strongly expressed in endomesodermal and neural ectodermal tissues (Fig. 1a7, a8). This result shows that *Smo* is expressed both maternally and zygotically in amphioxus embryos. This is different from the *Hh* gene, which shows zygotic but no maternal expression (Fig. 1b1-b8) [23]. *Hh* expression was first detected at G3 stage. The expression of *Hh* at the L0 stage (the mouth had just opened) was confined to the preoral pit and pharyngeal endoderm (Fig. 1b9, b10). From this, we speculate that zygotic mutation of *Smo* may not affect the early development of amphioxus embryos (e.g., left-right pattering) but disturbs late developmental events (e.g., mouth formation). We therefore anticipate that if Hh signal regulates mouth formation directly in the amphioxus, loss of Hh activation by *Smo* knockout in the amphioxus could lead to the failure of mouth formation. It would, however, not affect the development of left-right asymmetry, which can answer the question we raised at the beginning. To test this hypothesis, we generated *Smo* gene mutants using the TALEN method as previously described [19].

**Fig. 2:**
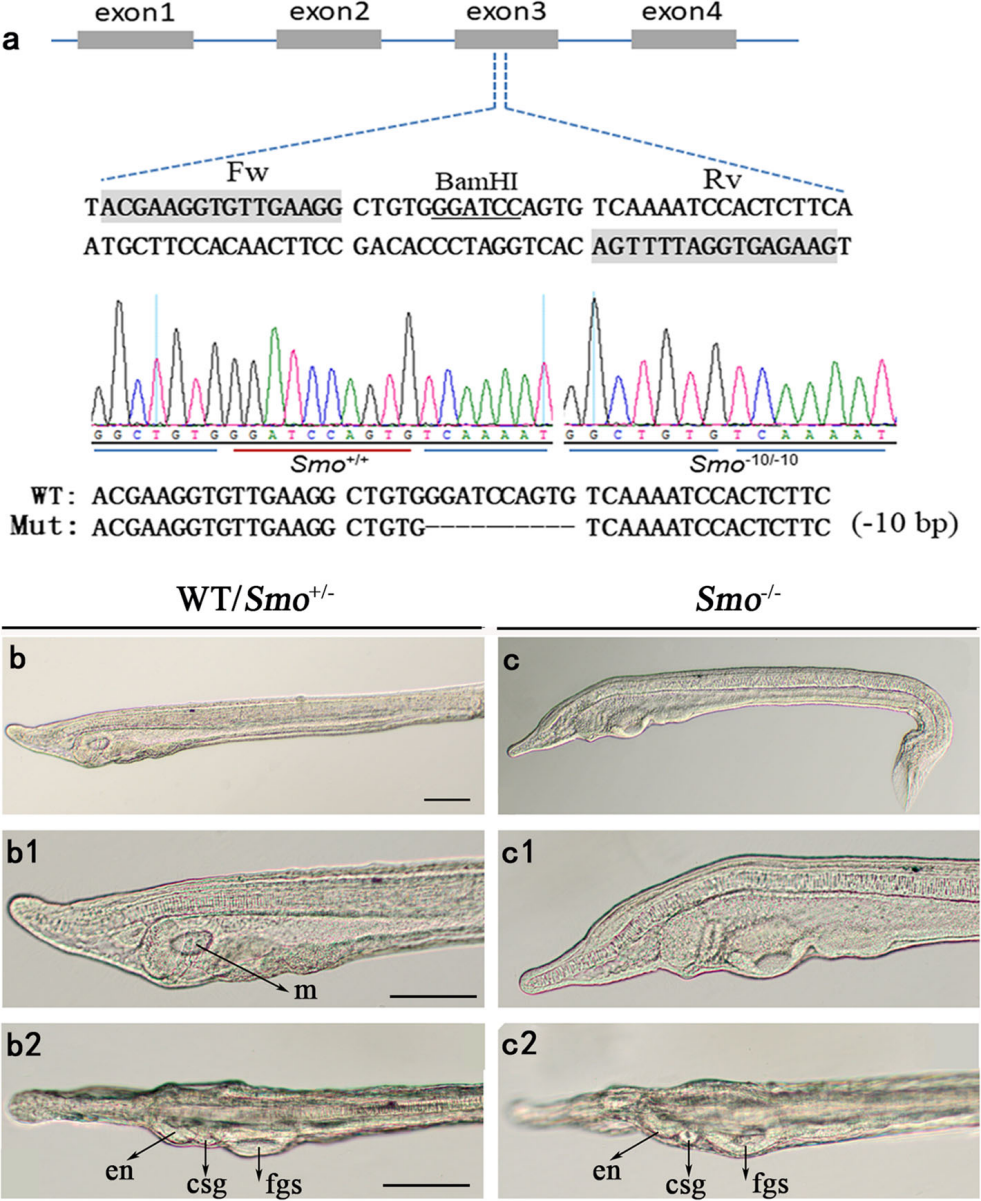
Hh signaling is necessary for mouth opening in the amphioxus. a: Information on the *Smo* TALEN target site and the sequencing results of wild-type and *Smo* mutant embryos. Binding sites for the TALEN pairs [forward (Fw) and reverse (Rv)] used in this study are highlighted in gray. The *BamH*I site in the spacer is underlined. b: phenotypes of wild-type and *Smo*^+/−^ embryos. c: phenotypes of *Smo* mutant embryos. The mouth present in wild-type and *Smo*^+/−^ heterozygotes is lost in *Smo*^−/−^ homozygous mutants, while the other pharyngeal organs, including the endostyle, club-shaped gland and first gill slit, are unaffected, m, mouth; en, endostyle; csg, club-shaped gland; fgs, first gill slit; scale bar 100 μm

**Fig. 3:**
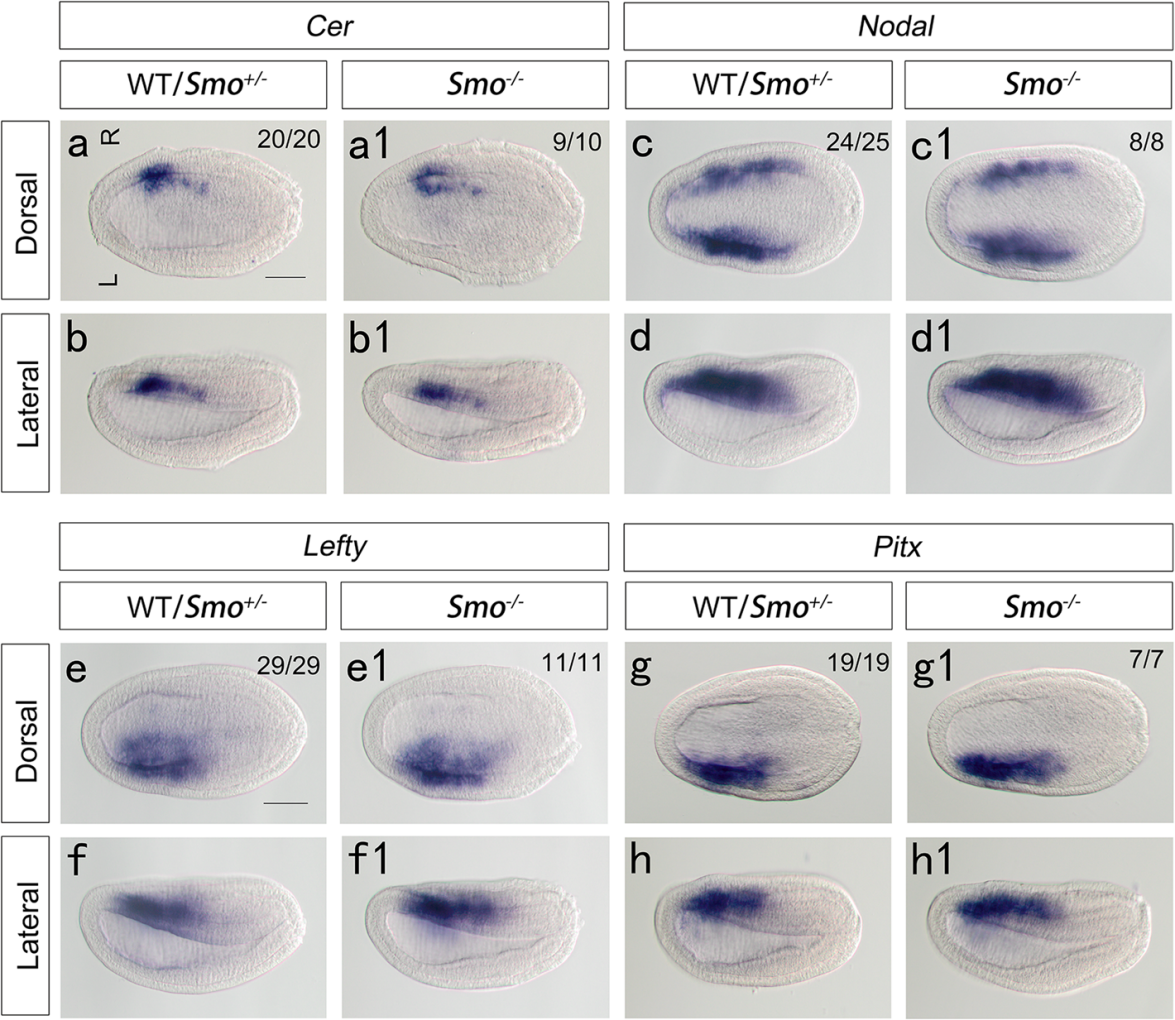
Expression of left-right regulatory genes in *Smo* mutant embryos. a, b: In the wild-type and *Smo*^*+/−*^ embryos, *Cer* is expressed mainly in the right paraxial mesoderm. a1, b1: *Cer* expression is unaffected in *Smo* mutant embryos. c, d: *Nodal* exhibits an L > R pattern in WT/*Smo*^*+/−*^ amphioxus at the early neurula stage. c1, d1: The asymmetrical expression of Nodal is unaffected in *Smo* mutant embryos. e, f: *Lefty* is expressed on the left side in WT/*Smo*^+/−^ embryos at early neurula stage; e1, f1: *Smo* knockout has no effect on *Lefty* expression; g, h: *Pitx* is expressed on the left side of WT/*Smo*^+/−^ embryos, *Smo* knockout has no effect on *Pitx* expression. Anterior to the left; L, left side; R, right side; Scale bar, 50 μm; Numbers in the top right corner of a panel show the number of times the phenotype depicted was seen out of the total number of embryos from that genotype analyzed

We constructed two pairs of TALENs targeting the coding sequence of the amphioxus *Smo* gene, namely, TALEN1 and TALEN2. TALEN mRNAs were injected into amphioxus embryos, and the somatic mutation frequencies were approximately 100% (TALEN1) and 50% (TALEN2) (Fig. S1). Because of the high mutant efficiency, TALEN1 mRNA-injected embryos cannot survive to adulthood to generate mosaic F0 animals. In this study, TALEN2 mRNA-injected embryos were raised to adulthood, and one of them carrying mutations at the target site in its germline was crossed with wild-type amphioxus. When F1 progenies developed to adulthood, we identified their genotypes one by one. Heterozygotes (*Smo*^+/−^) with a 10 bp deletion (Fig. 2a) were further used to obtain homozygous mutants. This mutation could induce an open reading frame (ORF) shift and thus generate a truncated protein with no functions. Next, we carefully examined the morphological features of the *Smo*^−/−^ mutant at the larval stage. Phenotypic examination revealed that the *Smo*^−/−^ mutants showed curved tails and no mouth opening (Fig. 2c), similar to *Hh* mutants [14]. However, in contrast to *Hh* mutants, *Smo* mutants developed a normal asymmetric arrangement of pharyngeal organs, including the preoral pit, endostyle and club-shaped gland (Fig. 2c1, c2). To verify that *Smo* knockout has no effect on left-right patterning in the amphioxus, we examined the expression patterns of left-right regulatory genes (including *Cer*, *Nodal*, *Lefty* and *Pitx*) in *Smo* mutants at the N1 neurula stage. At this stage, left-right patterning has already started, and the left-right regulatory genes exhibit an asymmetric expression pattern [10, 20, 24–26]. The results showed that in either wild type, *Smo*^+/−^ or *Smo*^−/−^ embryos, *Cer* was expressed on the right side, *Nodal* exhibited an L > R expression pattern, and *Lefty* and *Pitx* were expressed on the left side (Fig. 3). This result indicated that loss of Hh activation by *Smo* knockout had no effect on left-right patterning in the amphioxus. From these data and our previous findings [14], we conclude that the Hh signal is necessary for amphioxus mouth formation and that the Hh-mediated regulation of mouth development is specific to the mouth and independent of early morphogenetic defects of abnormal left-right patterning.

**Fig. 4:**
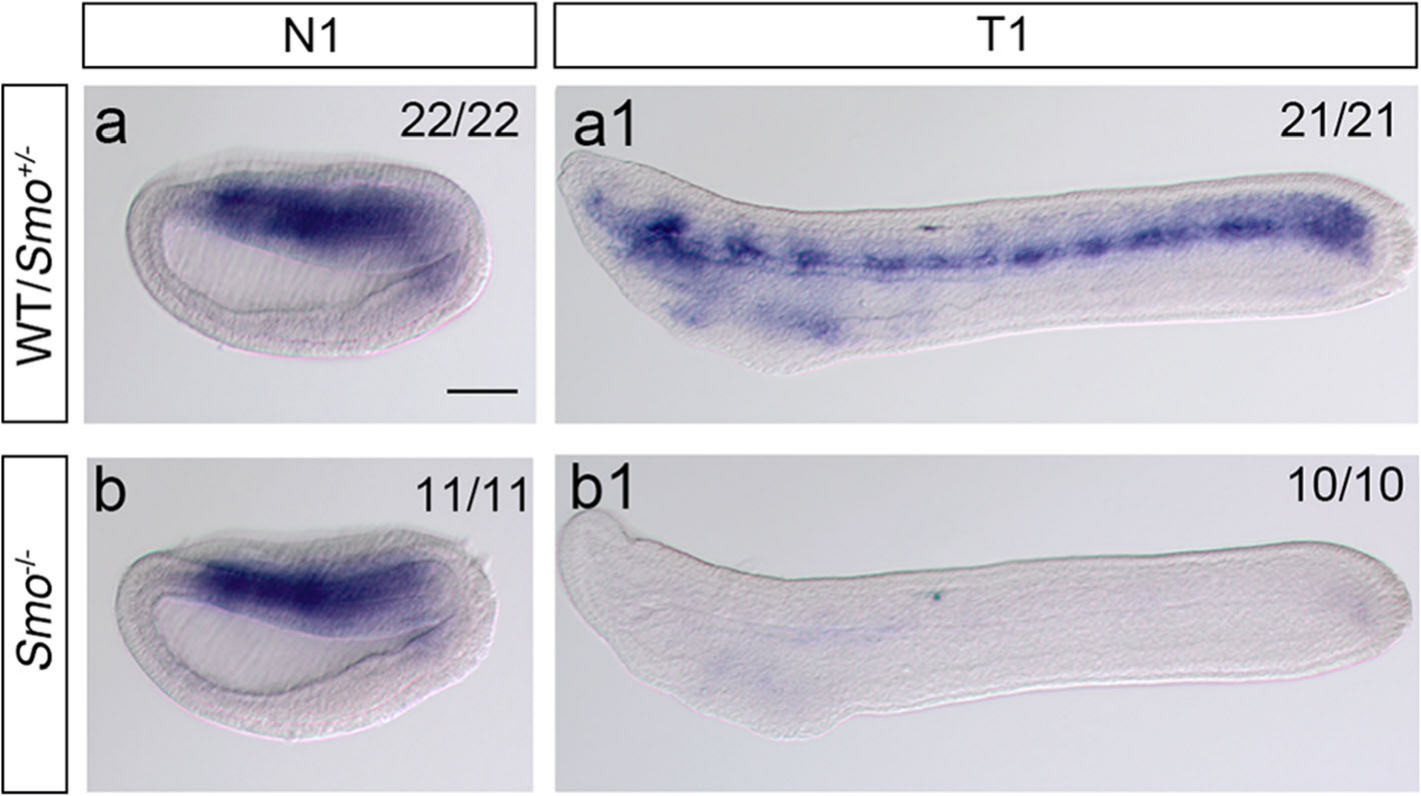
*Ptch* expression in *Smo* mutant embryos. *Ptch* expression was visualized by in situ hybridization, and all embryos were placed anterior to the left; a, b: *Ptch* expression in WT or *Smo*^*+/−*^ embryos; a1, b1: *Ptch* expression in *Smo*^*−/−*^ embryos. *Smo* gene knockout has no effect on *Ptch* expression at the N1 stage but results in diminished *Ptch* expression at the T1 stage. N1, early neurula stage; T1, tail bud stage before mouth opening; scale bar, 100 μm

To confirm that the inability of *Smo* knockout to affect left-right patterning is caused by its strong maternal expression, we next examined the expression of *Ptch* in *Smo*^−/−^ embryos at the early neurula stage (N1 stage) and tail bud stage (T1 stage) just before mouth opening. *Ptch* is the direct target of the Hh signaling pathway in vertebrates and the amphioxus [27–29], and thus, its expression can reflect the activity of Hh signaling. The results showed that in wild-type and *Smo*^+/−^ embryos, *Ptch* was expressed mainly on the dorsal endoderm at the N1 stage and somatic mesoderm and pharyngeal region at the T1 stage (Fig. 4a, a1). *Smo* knockout had no effect on *Ptch* expression at the early neurula stage (Fig. 4a, b) but diminished *Ptch* expression at the T1 stage (Fig. 4a1, b1). This result indicated that zygotic mutation of *Smo* had no effect on the activation of Hh signaling at early stage, at least before early neurula stage, showing that the inability of the *Smo* mutant to affect Hh signaling activation is due to *Smo*’s maternal expression.

**Fig. 5:**
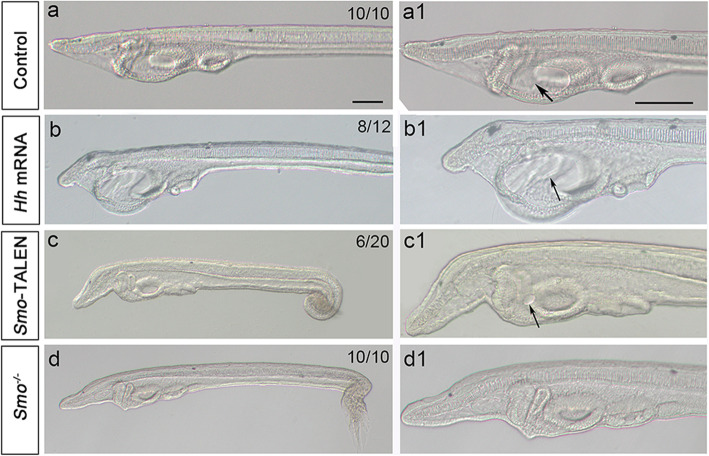
Hedgehog perturbation affects the size of amphioxus mouth. a, a1: Left lateral view of L1 stage larvae focused on the left-sided mouth opening in control embryos (black arrow), Scale bar, 100 μm. b, b1:*Hh* mRNA injection results in a dramatic increase in mouth size. c, c1: *Smo*-TALEN mRNA injection results in a small mouth in the amphioxus. d, d1: *Smo* gene knockout leads to a complete loss of mouth opening. Numbers in the top right corner of a panel show the number of times the phenotype was observed in the total number of embryos examined

In *Xenopus*, Hh signaling is required to regulate primary mouth size*,* loss of Hh activation results in a small or absent primary mouth, and increased Hh activation leads to a larger mouth [11]. To determine whether Hh signaling regulates mouth size in the amphioxus, we next examined mouth development in *Hh* mRNA-injected embryos. We previously showed that *Hh* mRNA injection effectively upregulates Hh signaling [29]. Compared with control embryos, *Hh* mRNA injection caused a dramatic increase in mouth size (Fig. 5b, b1). Conversely, in *Smo*-TALEN mRNA-injected (*Smo* gene knockdown) embryos, 30% (6/20) showed a small mouth phenotype (Fig. 5c, c1), and *Smo* homozygous mutants showed a complete loss of mouth opening (Fig. 5d, d1). Together, these data demonstrate that Hh signaling regulates amphioxus mouth development in a similar way as in vertebrates.

**Fig. 6:**
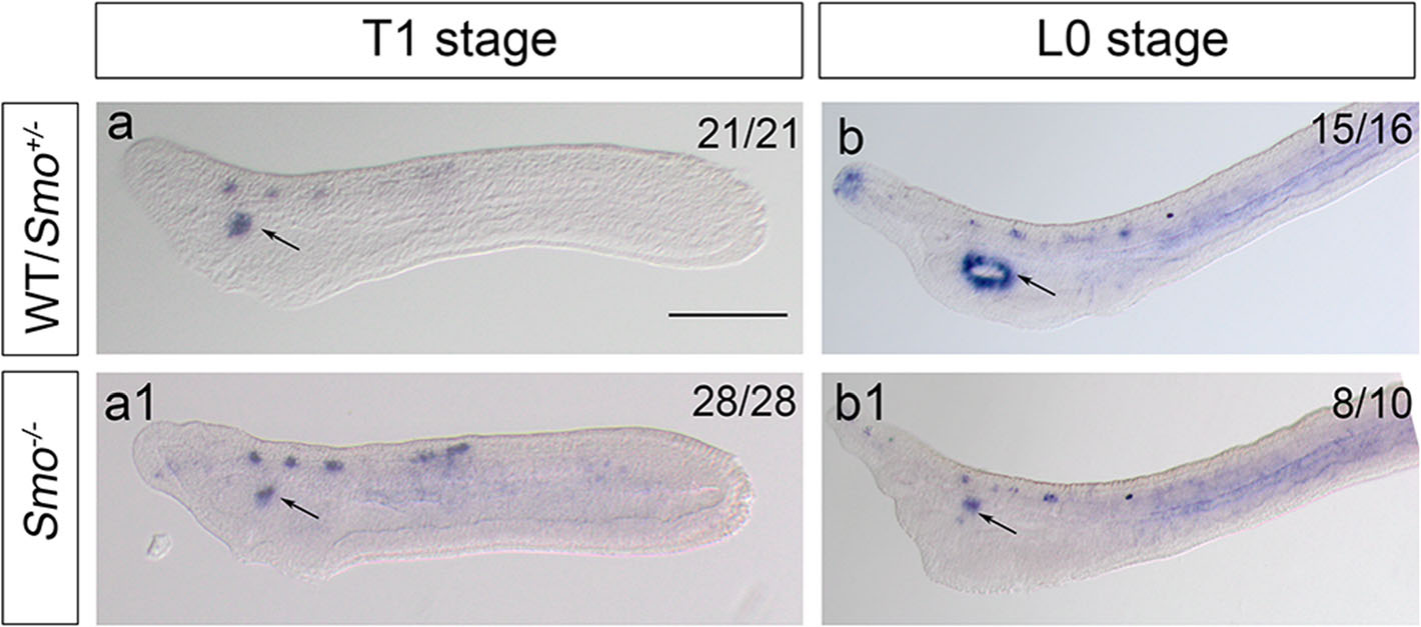
*Pou4* expression during mouth development in the amphioxus. Images of *B. floridae* at the tail bud stage (T1 stage) and the open mouth stage (L0 stage) from the left lateral view. Black arrows mark the mouth region, scale bar, 100 μm. a, a1: *Pou4* is expressed focally in the regions destined to form the mouth (a), *Smo* gene knockout has no effect on *Pou4* expression (a1). b, b1: *Pou4* is expressed at the margin of the mouth at the L0 stage. *Smo* knockout results in diminished *Pou4* expression at the mouth margin with the complete loss of mouth opening

Having shown that the Hh signal is specific to regulate mouth formation and modulate mouth size in the amphioxus, we next tested whether Hh activation functions throughout mouth development or at a specific stage. The developmental process of the amphioxus mouth has been elucidated in previous study, including the formation of OMV and mouth perforation [1, 2], and this process can be visualized by *Pou4* expression. *Pou4* is expressed dynamically during mouth development: at the early larval stage, *Pou4* is expressed in the oral region, including the OMV, and then at the margin of the mouth during perforation [30]. To determine the functional stage of Hh signaling during amphioxus mouth development, we examined the expression of *Pou4* in *Smo* mutant embryos. The results showed that in wild-type embryos, *Pou4* was expressed in the primordial oral site before mouth opening and then at the margin of the mouth after the mouth had opened (Fig. 6a, b). Loss of Hh activation by *Smo* gene knockout did not affect *Pou4* expression at the T1 stage (before mouth opening) (Fig. 6a1) but diminished the expression of *Pou4* at the margin of the mouth with the complete loss of mouth opening (Fig. 6b1). This result showed that the initial specification of the mouth in the amphioxus may not depend on Hh signaling. To verify this, we also examined the expression of *Pax2/5/8* at the primordial oral site before mouth opening (T1 stage) [31]. In agreement with *Pou4* expression at the T1 stage, *Pax*2/5/8 expression was not affected at the T1 stage in embryos with loss of Hh activation (Fig. S2). Taken together, these results indicated that the initial specification of the mouth may not depend on Hh activation; however, the perforation of the mouth is probably controlled by Hh signaling.

In vertebrates, oral perforation is characterized by dissolution of the ectoderm and endoderm, and Hh signaling plays a key role in this process [13]. In the amphioxus, the molecular mechanisms regulating mouth formation remain rather scarce, especially for perforation. In this study, we showed that Hh signaling is necessary for the development of the mouth in amphioxus larvae probably through controlling perforation. Until now, it has been uncertain whether amphioxus mouth penetration results from fusion of the ectoderm and endoderm like that in vertebrates [1]. A Study in *Xenopus* showed that Wnt antagonists Frzb1 and Crescent regulate mouth perforation in the developing primary mouth [32]. During primary mouth formation, Frzb1 and Crescent inhibit Wnt signaling, which prevents the synthesis of the proteins Laminin and Fibronectin, which are essential for basement membrane dissolution [32]. Although no basal lamia around the OMV was found during amphioxus mouth formation [2], we showed that loss of Hh activation diminished *Frzb1* expression in the mouth region (Fig. S3). Therefore, it is tempting to hypothesize that Wnt signaling may exert important roles during amphioxus mouth perforation, similar to that in vertebrates. Further studies are needed to investigate the relationship of Hh and Wnt pathway during amphioxus mouth perforation and to clarify whether the amphioxus mouth is homologous to that of vertebrates.

## Conclusion

In this study, we showed that *Smo* is expressed maternally and zygotically in *B. floridae*, which is different from the previous report that *Smo* expression begins at the blastula stage in *Branchiostoma belcheri* [33]. Thanks to the maternal expression of *Smo*, loss of Hh activation by *Smo* knockout did not affect amphioxus left-right asymmetric development but resulted in a complete loss of mouth formation, showing that Hh-mediated regulation of mouth development is specific to the mouth and can be uncoupled from early defects of impaired left-right patterning. Our results provide the first demonstration of a role for Hh signaling in amphioxus mouth development. The unusual location of the amphioxus mouth has puzzled researchers for more than 100 years, and various hypotheses have been proposed to explain the evolutionary history of the amphioxus mouth. Our results significantly advance our understanding of amphioxus mouth development and provide a new direction for researchers to further explore the genetic regulation of mouth development. Moreover, our results pinpoint a novel role for Hh signaling during amphioxus embryo development.

## Supplementary Information

**Additional file 1: Fig. S1** TALEN-mediated genome editing at amphioxus *Smo* locus. a: F0 Mutagenesis efficiency of two *Smo*-TALEN targets (estimated as percentages of uncut PCR products), M: DNA marker, WT shows PCR products amplified from the genomic DNA extracted from wild-type embryos and treated with restriction endonucleases; TALEN1 shows the efficiency of target 1, TALEN2 shows the efficiency of target 2 (used in this study). White arrows mark the uncut bands; **b**: Mutagenesis efficiency of F0 (1# male) generation gamete, PCR: PCR product without enzyme digestion; **c**: Sampling test of F1 generation, numbers indicate the number of individuals. Number 2 is heterozygote

**Additional file 2: Fig. S2** *Pax2/5/8* expression in *Smo* mutant embryos. *Pax2/5/8* expression was visualized by in situ hybridization. All embryos were placed with the head to the left, arrows mark the regions destined to form the mouth, scale bar, 100 μm; a, left lateral view; b, dorsal view. *Smo* knockout has no effect on *Pax*2/5/8 expression at the region where the mouth will form.

**Additional file 3: Fig. S3** *Frzb1* expression in *Hh* mutant embryos. *Frzb1* expression at the L0 stage was visualized by in situ hybridization. All embryos were placed with the head to the left, and the arrow marks the mouth region. Scale bar, 100 μm. Loss of Hh activation diminished *Frzb1* expression at the mouth region.

## Data Availability

The datasets used and/or analyzed during the current study are available from the corresponding author on reasonable request.

## Abbreviations

Hh: Hedgehog
LR: Left-right
Smo: Smoothened
OMV: Oral mesovesicle
Ptch: Patched
