# Supplementary figures and images for: Hedgehog signaling controls mouth opening in the amphioxus
Source: Zoological Lett. 2021 Dec 24;7:16. doi: 10.1186/s40851-021-00186-8 (PMC8709984; doi:10.1186/s40851-021-00186-8)

## Slide 1
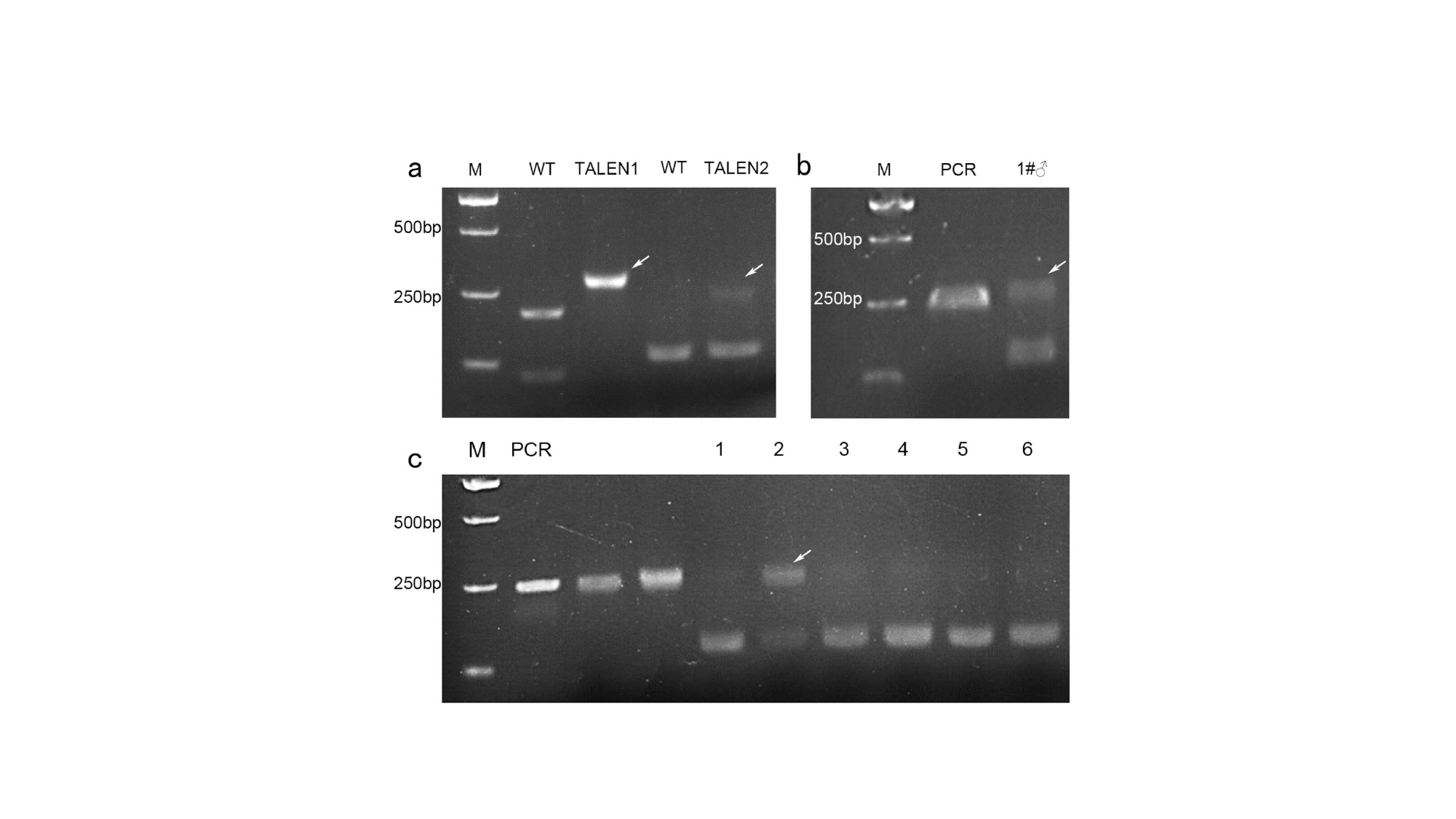

Supplement: Supplementary file 1 — Additional file 1: Fig. S1 TALEN-mediated genome editing at amphioxus Smo locus. a: F0 Mutagenesis efficiency of two Smo-TALEN targets (estimated as percentages of uncut PCR products), M: DNA marker, WT shows PCR products amplified from the genomic DNA extracted from wild-type embryos and treated with restriction endonucleases; TALEN1 shows the efficiency of target 1, TALEN2 shows the efficiency of target 2 (used in this study). White arrows mark the uncut bands; b: Mutagenesis efficiency of F0 (1# male) generation gamete, PCR: PCR product without enzyme digestion; c: Sampling test of F1 generation, numbers indicate the number of individuals. Number 2 is heterozygote [file 40851_2021_186_MOESM1_ESM.pptx]

## Slide 1
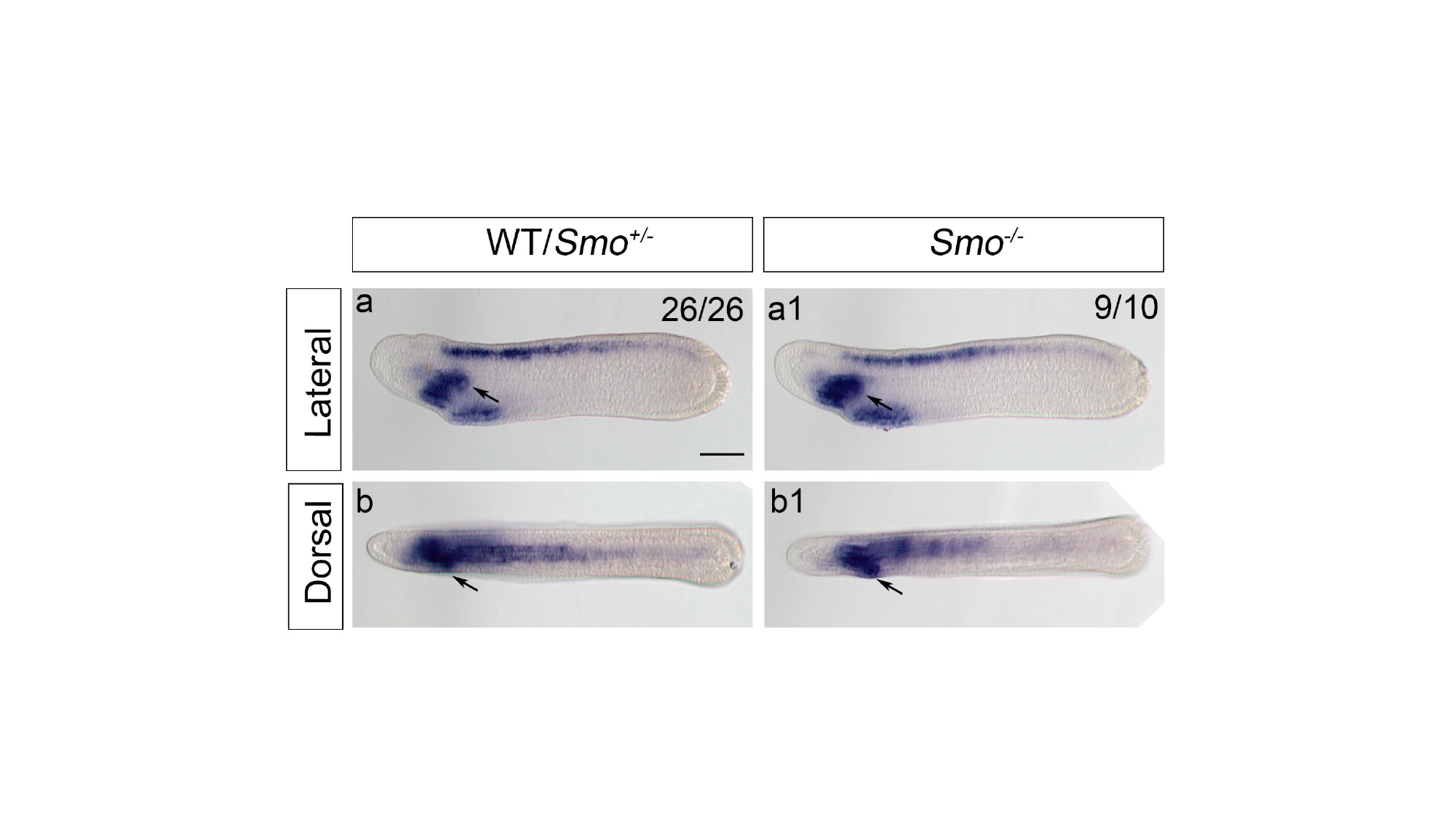

Supplement: Supplementary file 2 — Additional file 2: Fig. S2 Pax2/5/8 expression in Smo mutant embryos. Pax2/5/8 expression was visualized by in situ hybridization. All embryos were placed with the head to the left, arrows mark the regions destined to form the mouth, scale bar, 100 μm; a, left lateral view; b, dorsal view. Smo knockout has no effect on Pax2/5/8 expression at the region where the mouth will form. [file 40851_2021_186_MOESM2_ESM.pptx]

## Slide 1
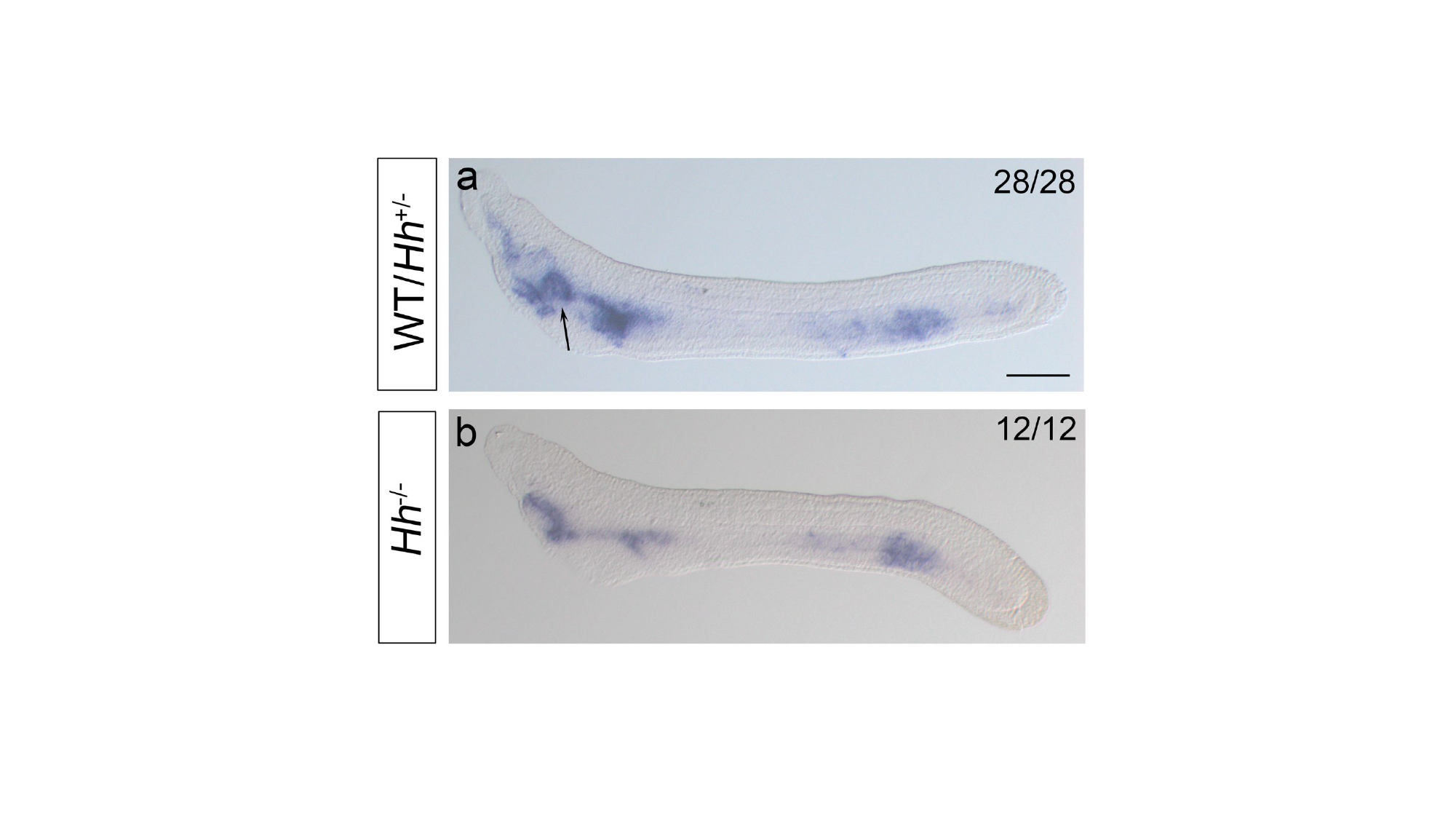

Supplement: Supplementary file 3 — Additional file 3: Fig. S3 Frzb1 expression in Hh mutant embryos. Frzb1 expression at the L0 stage was visualized by in situ hybridization. All embryos were placed with the head to the left, and the arrow marks the mouth region. Scale bar, 100 μm. Loss of Hh activation diminished Frzb1 expression at the mouth region. [file 40851_2021_186_MOESM3_ESM.pptx]
